# Visualization of blood supply route to the reconstructed stomach by indocyanine green fluorescence imaging during esophagectomy

**DOI:** 10.1186/1471-2342-14-18

**Published:** 2014-05-22

**Authors:** Yasushi Rino, Norio Yukawa, Tsutomu Sato, Naoto Yamamoto, Hiroshi Tamagawa, Shinichi Hasegawa, Takashi Oshima, Takaki Yoshikawa, Munetaka Masuda, Toshio Imada

**Affiliations:** 1 Department of Surgery, Yokohama City University, 3-9, Fukuura, Kanazawa-ku, Yokohama city 236-0004, Japan; 2 Gastroenterological Center, Medical Center, Yokohama City University, Yokohama, Japan; 3 Department of Gastrointestinal Surgery, Kanagawa Cancer Center, Yokohama, Japan; 4 Saiseikai Yokohama Nanbu Hospital, Yokohama, Japan

**Keywords:** ICG, Esophageal cancer, Blood supply route, Splenic hiatal vessels

## Abstract

**Background:**

Ensuring an adequate blood supply is essential to the safe performance of an anastomosis during esophagectomy and the prevention of anastomotic leakage. Recently, indocyanine green (ICG) fluorescence imaging has been used to visualize the blood supply when anastomosis is performed in vascular surgery. We used ICG fluorescence imaging to visualize the blood supply for reconstruction during esophagectomy.

**Methods:**

Since January 2009, we have performed ICG fluorescence imaging in 33 patients with thoracic esophageal cancer who underwent thoracic esophagectomy. After pulling up the reconstructed stomach, 2.5 mg of ICG was injected as a bolus. ICG fluorescence imaging was performed with a near-infrared camera, and the images were recorded.

**Results:**

ICG fluorescence was easily detected in all patients 1 min after injection. Vascular networks were well visualized in the gastric wall and omentum. The blood supply route was located in the greater omentum beside the splenic hilum in 22 (66.7%) of the 33 patients.

**Conclusions:**

ICG fluorescence can be used to evaluate the blood supply to the reconstructed stomach in patients undergoing esophagectomy for esophageal cancer. On ICG fluorescence imaging, the splenic hiatal vessels were the major blood supply for the anastomosis in most patients.

## Background

The stomach is most widely used to reconstruct the alimentary tract after esophagectomy for esophageal cancer. Ensuring an adequate blood supply is essential to the safe performance of an anastomosis after esophagectomy and the prevention of anastomotic leakage.

The arterial blood supply to the stomach is derived predominantly from the celiac axis, although intramural anastomoses with vessels of other origins exist at the two ends of the stomach. The left gastric artery arises directly from the celiac axis. The splenic artery gives rise to the short gastric arteries as well as the left gastroepiploic artery and may occasionally give rise to a posterior gastric artery. The hepatic artery gives rise to the right gastric artery and the gastroduodenal artery, which in turn give rise to the right gastroepiploic artery [1].

Many institutions use the stomach for reconstruction of the alimentary tract after esophagectomy. Generally, the right gastroepiploic artery and right gastric artery are preserved to supply blood to the gastric tube. Only the right gastroepiploic artery is preserved when a gastric tube is constructed from the greater curvature. The right gastroepiploic artery thus serves as the main source of blood to the reconstructed stomach.

To assess blood supply to reconstructed organs, laser Doppler flowmetry has been used, but the reliability of the results has been questioned [2,3]. Recently, indocyanine green (ICG) fluorescence imaging has been used to detect sentinel lymph nodes during surgery for breast cancer, gastric cancer, and colorectal cancer, as well as to visualize the blood supply after anastomosis during vascular surgery [4-9]. We have used ICG fluorescence imaging since January 2009 to visualize the blood supply of reconstructed organs during esophagectomy. To our knowledge, blood flow routes of the reconstructed stomach have not been reported previously. This study was performed to evaluate blood supply routes of the reconstructed stomach by ICG fluorescence imaging.

## Methods

### Patient characteristics

This study was approved by the institutional ethical committee. Research carried out on humans must be in compliance with the Helsinki Declaration. Informed consent was obtained from all patients. We studied 33 patients (29 men, 4 women; mean age, 67.8 years) who had a preoperative diagnosis of thoracic esophageal cancer. We evaluated blood flow of the reconstructed stomach with a photodynamic eye (PDE) after ICG injection and the incidence of anastomotic leakage and stenosis.

### ICG imaging procedure

We made a 4-cm-wide gastric tube with an autosuture device. Five to six 6 cm-cartridges of an autosuture device were used. Seromuscular suture was done for all patients to avoid contact between stapler and the lung. The right gastric vessels and right gastroepiploic vessels were preserved. After preparation, we performed ICG fluorescence imaging of the gastric tube. We recorded ICG fluorescence images of the right gastric artery, right gastroepiploic artery, vascular networks within the stomach wall at the intramuscular, submucosal, and mucosal levels, and vascular networks within the omentum. After pulling up the reconstructed stomach, 2.5 mg of ICG dye (Diagnogreen; Daiichi Sankyo Company, Limited, Tokyo, Japan) was injected as a bolus. Then, ICG fluorescence imaging was performed with a near-infrared camera system (PDE; Hamamatsu Photonics K.K, Hamamatsu, Japan), and the images were recorded. In brief, images were obtained with a charge-coupled device (CCD) camera, using a light-emitting diode with a wavelength of 760 nm as the light source and a filter to eliminate light of wavelengths below 820 nm before detection [10]. Images were sent to a digital video processor and then displayed on a monitor. We performed anastomosis of the esophagus to the good vascular network wall of the gastric tube in the thoracic cavity. Because our PDE cannot evaluate blood flow of the reconstructed stomach in the thoracic cavity, we did not evaluate blood flow around the anastomotic site after anastomosis.

## Results

**Figure 1:**
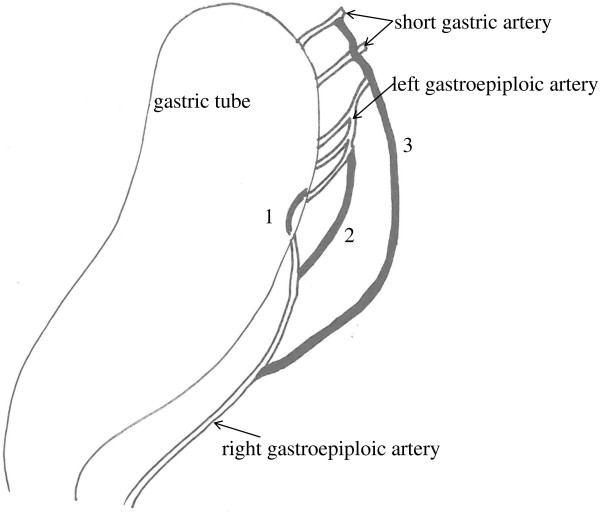
Schema of the categories on the basis of ICG imaging findings: 1; the gastric wall route, 2; the greater curvature route, and 3; the omentum and splenic hiatal route.

**Figure 2:**
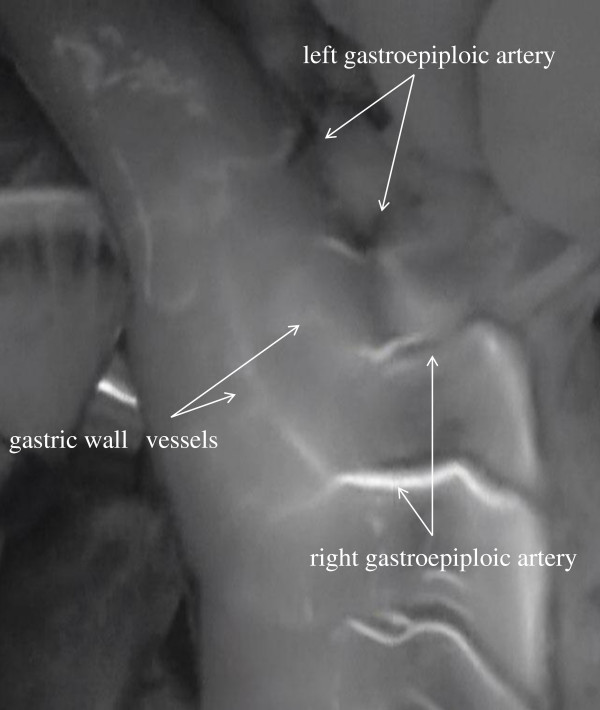
The PDE system showed blood flow from the right gastroepiploic artery, gastric wall vessels, and left gastroepiploic artery of the reconstructed stomach.

**Figure 3:**
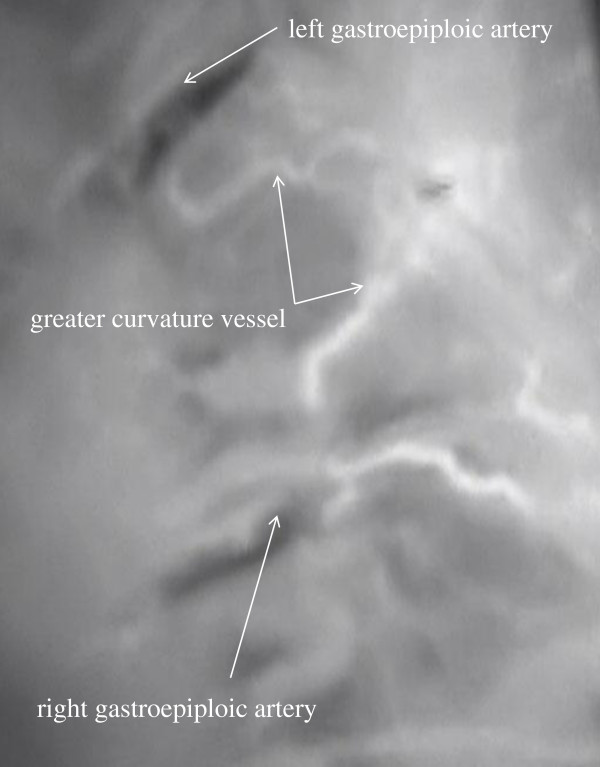
The PDE system showed blood flow from the right gastroepiploic artery, left gastroepiploic artery, and greater curvature vessels of the reconstructed stomach.

**Figure 4:**
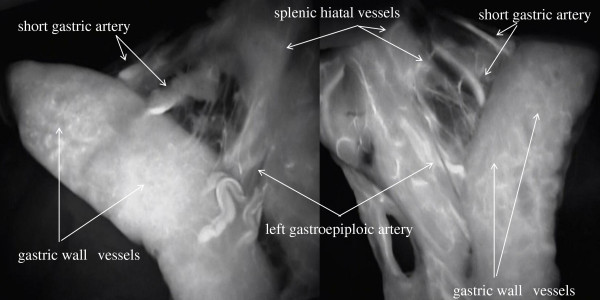
The PDE system showed blood flow from the omentum vessels, left gastroepiploic artery, splenic hiatal vessels, short gastric artery, and gastric wall vessels to the top of the reconstructed stomach.

Blood flow routes in the 33 patients with esophageal cancer were classified into categories on the basis of ICG imaging findings: the gastric wall route, the greater curvature route, and the omentum and splenic hiatal route (Figure 1). The gastric wall route was found in 13 patients and characterized by large blood vessels in the gastric wall. The PDE system showed blood flow from right gastroepiploic artery, gastric wall vessels, and left gastroepiploic artery to the top of the reconstructed stomach (Figure 2). The greater curvature route was present in 11 patients and characterized by blood vessels in the greater omentum. The PDE system showed blood flow from the right gastroepiploic artery, left gastroepiploic artery, and vessels beside the greater curvature to the top of the reconstructed stomach (Figure 3). The omentum and beside splenic hilum route was found in 22 patients. Blood vessels ran in the greater omentum beside the splenic hilum. PDE showed blood flow from right gastroepiploic artery, omentum vessels, left gastroepiploic artery, splenic hiatal vessels, short gastric artery, and gastric wall vessels to the top of the reconstructed stomach (Figure 4). The “splenic hiatal route” was present in 66.7% of the patients and was formed by large vessels or networks of small vessels. Two blood flow routes were found in 12 (36.4%) of the 33 patients: 6 (50.0%) of 12 patients with the “splenic hiatal route” and the greater curvature route, and 6 (50.0%) of 12 patients with the “splenic hiatal route” and the gastric wall route.

Anastomotic leakage occurred in 5 (15.2%) of the 33 patients: 4 (18.2%) of 22 patients with the “splenic hiatal route” and 1 (9.1%) of 11 patients without the “splenic hiatal route”. Anastomotic stenosis occurred in 1 (3.0%) of 33 patients without “splenic hiatal route”.

## Discussion

Intravital fluorescence-based optical imaging has inherent advantages owing to its high sensitivity, fast feedback, multiplexing, and absence of ionizing radiation. Several fluorescence imaging agents with emission <1000 nm, such as ICG, have been used to image microvasculature and lymph nodes. However, minimal penetration depth with low feature fidelity has largely limited their further clinical applications. Fluorescence imaging in the second near-infrared region (NIR-II, 1000–1350 nm) is more desirable than visible (450–700 nm) and traditional NIR-I imaging (700–950 nm) owing to greatly reduced photon absorption and scattering by tissues, as well as negligible tissue autofluorescence, which promises high-fidelity imaging of deeper tissues and organs. As compared with ICG (NIR-I emission peak at 835 nm), Ag_2_S quantum dots (QDs) (NIR-II emission peak at 1200 nm) maintained higher image fidelity and integrity, effects attributable to minimized scattering in the NIR-II window. Ag_2_S QDs are promising NIR-II fluorescent nanoprobes that could be useful in surgical procedures such as sentinel lymph node (SLN) dissection, as well in assessing the blood supply in tissues and organs and screening for anti-angiogenic drugs [11]. Measurement of tissue oxygen levels, laser Doppler flowmetry, and laser fluorescence angiography (LFA) are some of the more reliable methods that have been used to evaluate tissue perfusion [12-16]. LFA is a method that may significantly reduce the rate of severe complications in colorectal surgery, as well as shorten the length of hospital stay [17]. However, we do no have Ag_2_S QDs (NIR-II emission peak at 1200 nm) or LFA in our hospital.

Some authors have reported that PDE is useful for evaluating the blood supply of reconstructed organs [18-20]. To our knowledge, however, ICG imaging has not been used previously to study the blood supply route of the gastric conduit in patients undergoing esophagectomy for cancer of the thoracic esophagus. We evaluated blood flow of the reconstructed stomach with the use of a PDE system after ICG injection. Splenic hiatal vessels were detected in a high proportion of patients. Preservation of this “splenic hiatal route” would maintain blood flow to the top of the reconstructed stomach. However, preservation of the “splenic hiatal route” does not ensure the prevention of anastomotic leakage. We performed anastomosis of the esophagus to the good vascular network wall of the gastric tube in the thoracic cavity. After this report, we performed anastomosis of the esophagus to the grater curvature avascular area of the gastric tube for three esophageal cancer patients. Anastomotic leakage and stenosis were not experienced. We presume that our previous anastomotic method break the good gastric tube vascular network. Besides the blood supply, other risk factors for anastomotic leakage include venous return, congestion, and tension on the anastomotic site. PDE does not have the ability to evaluate venous return or gastric-tube congestion. LFA is a method that may significantly reduce not only the rate of severe complications in colorectal surgery but also the length of hospital stay [17]. We believe that the use of LFA will substantially reduce the rate of complications in esophageal surgery.

## Conclusion

The blood vessels were difficult to macroscopically confirm in patients with excessive adipose tissue without PDE system, making preservation of the splenic hiatal vessels challenging.

## Competing interest

There are no financial or other relations that could lead to a conflict of interest.

There are any non-financial competing interests (political, personal, religious, ideological, academic, intellectual, commercial or any other) to declare in relation to this manuscript.

## Authors’ contributions

YR was the lead author and surgeon for all of the patients. NY and TS contributed to the patients and information on the patients. NY, HT, and SH were the co-surgeons of the patients. TO, and TY gathered information and contributed to writing the paper. MM and TI reviewed paper and surgical technique. All authors read and approved the final manuscript.

## Pre-publication history

The pre-publication history for this paper can be accessed here:

http://www.biomedcentral.com/1471-2342/14/18/prepub
